# A Death during the Administration of Chloroform

**Published:** 1880-07

**Authors:** 


### A DEATH DURING THE ADMINISTRATION OF CHLOROFORM.

The following account of a case of death, on May 18th, from the effects of chloroform during its administration previous to an operation, and which excited an unusual amount of interest among both the profession and the general public, is condensed from the very full account in the Buffalo *Courier* of the testimony given at the coroner's inquest.

The deceased was a man of about 40 years; the operation was for stone in the bladder, though one physician, who examined him five or six weeks previously, found a large stone in the urethra, where one was found at the autopsy.

Patient's health was somewhat affected by his disease; he was nervous and anxious in regard to the operation, and expressed fear of taking chloroform, and requested ether; this request was made to others, but not to the operator. The heart was examined by the surgeon a day or two before the operation, and also by another physician several weeks before, both of whom declared the organ normal. The chloroform (which was Squibb's, and carefully examined by a chemist after the operation and pronounced pure) was first administered by the operator, and after about ten minutes handed to an assistant; it was poured from a small bottle, about thirty minims at a time, on to a folded napkin. Before becoming totally unconscious, the patient sprang from the table, and struggled violently with those holding him; suddenly his strength gave way, and he said he would get back on the table, then sank on to the floor and ceased to breathe; artificial respiration was immediately begun, and continued half an hour without avail. It appeared that both principal and assistant were accustomed to give chloroform, and that the drug was administered with care. An autopsy was made, and the brain and organs, except the heart, found to be healthy; "the muscles of the heart seemed a little pale; on pressing, it pitted and tore as easily as wet blotting-paper; there was fatty degeneration of the heart; the muscular fibres were filled with what is called fatty granules; there was slight disease of both valves, but in all probability not sufficient to be detected during life." It was in evidence by another physician, although not reported so in the paper, that the condition was one of fatty infiltration, but nothing was said by this examiner of degeneration; the pulse was strong and full a few minutes before the patient sprang from the table.

The following is the verdict:

That the said Frederick J. Turner came to his death from fatty degeneration of the heart, while inhaling the vapor of chloroform preparatory to undergoing a surgical operation solicited on his part. That the inhalation of said vapor caused great excitement and muscular effort which, together with the fear of the operation, were the exciting causes of his death. The jury further find that the chloroform was a proper anæsthetic, and was administered in a careful and skillful manner, and that the attending physician took the necessary precaution to make a physical examination before giving the anæsthetic, and he and his assistants are exonerated from all censure.

